# Mental Health Impact of Mass Depopulation of Swine on Veterinarians During COVID-19 Infrastructure Breakdown

**DOI:** 10.3389/fvets.2022.842585

**Published:** 2022-04-05

**Authors:** Angela Baysinger, Lori R. Kogan

**Affiliations:** ^1^Veterinary and Consumer Affairs, Merck Animal Health, DeSoto, KS, United States; ^2^Department of Clinical Sciences, College of Veterinary Medicine and Biomedical Sciences, Colorado State University, Fort Collins, CO, United States

**Keywords:** mental health, depopulation, swine, COVID-19, well being, psychological distress

## Abstract

This study was designed to assess the mental health of swine veterinarians involved with mass depopulation events related to COVID-19 and compare them to swine veterinarians not involved in mass depopulation. Additionally, we assessed the well being, quality of life, psychological distress, burnout, and resilience in veterinarians who conducted depopulation events and the potential impact of depopulation methods on these factors. Finally, we identified coping methods utilized by swine veterinarians for improved well being. The study involved the distribution of an anonymous online survey, available December 2020 to January 2021, to swine veterinarians practicing in the United States. A total of 134 responses were analyzed. Stress related to the depopulation effort was predominantly an outcome of two factors: ethics of care (people and pigs) and perception of others (public, colleagues, family, friends, neighbors). Depopulation involvement was associated with burnout (p = 0.001). The depopulation method utilized significantly impacted depopulation distress (p = 0.007), perception of others (*p* < 0.001), and burnout (*p* < 0.001). Nearly one-third (29%) of all participants reported moderate levels of burnout. Based on these results, the call to action is to enhance the availability and visibility of existing mental health services and take necessary steps to destigmatize mental health. Additionally, it is critical to support the development of mental health programs for swine veterinarians through education, training, research, and transparent communication.

## Introduction

In the last 5 years, the well being of veterinarians has been prioritized and evaluated as a profession with significant mental health challenges (1–10). However, the veterinarians that practice swine veterinary medicine in the United States have not been evaluated as a subset of the profession. Additionally, a portion of the swine veterinarians' application and oversight of mass depopulation needed to be studied utilizing the non-participating swine veterinarians as a comparative cohort. Understanding the mental health baseline of swine veterinarians and the subsequent effect of mass depopulation can guide the veterinary profession for the future care of veterinarians in critical emergency situations.

The restriction of animal movement to slaughter started affecting U.S. swine producers in late March 2020 as packing plants started diagnosing COVID-19 in their worker populations (11). This unprecedented pork processing disruption can only be equated to the live animal movement restrictions experienced during previous disease eradication efforts related to foreign animal disease (FAD) (12). During FAD outbreaks, the animals that cannot be salvaged as food are strategically killed to relieve overcrowding or other deteriorating animal welfare situations. This process is called “Welfare Slaughter” (13), defined and quantified by Terry Whiting (14–16). The Canadian Food and Agriculture Emergency Response System (FAERS) defines this type of emergency as “an abnormal situation requiring prompt action beyond normal procedures to prevent injury or damage to people, plants, livestock, property or the environment (17). The American Veterinary Medical Association speaks to this issue within their guidelines on the depopulation of animals (18). Additionally, the American Association of Swine Veterinarians clarifies that depopulation is necessary for several situations, including market disruption that can negatively impact animal welfare (19–21).

Veterinarians are at the center of the emergency mass depopulation process, providing guidance and animal welfare oversight. The responsibility of euthanizing animals, under any circumstances, can create moral stress, often called the “caring-killing paradox,” as veterinarians struggle to balance their love for animals with the necessity to euthanize (22–24). Studies involving those whose work involves euthanizing animals have found that many of these individuals suffer from a form of post-traumatic stress disorder (PTSD) labeled perpetration-induced traumatic stress (PITS) (25, 26). PITS differs from PTSD because PTSD follows a life-threatening or terrifying experience. Alternately, PITS does not involve a direct threat to the individual; instead, it may threaten their ethical character; *per se*, their identity as a veterinarian who cares for animals yet must implement euthanasia or the death of that animal (16, 27, 28). This phenomenon, including its psychological and emotional impact, has pe rhaps been most studied in shelter animal caretakers (26, 29–31). Additionally, these adverse effects have been seen in farm caretakers conducting routine applications and farmers impacted by euthanizing animals during a foot and mouth disease outbreak (32–34). A study by Makita, focusing on the mental distress of field veterinarians in the 2010 foot and mouth disease outbreak in Japan, found similar negative effects (35).

Yet, veterinarians who applied mass depopulation methods during COVID-19 faced an unprecedented challenge, and it is unknown how the depopulation events may have impacted their psychological and emotional well being. Therefore, this study's goals were five-fold:

1) Assess the mental health of swine veterinarians compared to the U.S. veterinary population, including well being, quality of life (QOL), psychological distress, burnout, and resilience.
2) Identify coping methods utilized by swine veterinarians for improved well being.
3) Compare the mental health of swine veterinarians involved with mass depopulation events with swine veterinarians who were not involved in mass depopulation.
4) Measure the potential impact of depopulation methods on swine veterinarians' mental health.
5) Based on the study results, make recommendations for intervention strategies and supportive services to assist veterinarians in future mass depopulation events.

## Methodology

An anonymous online survey was created to evaluate swine veterinarians' experiences and perceptions regarding the COVID-19 depopulation event from April 2020 to June 2020. Researchers from Colorado State University and Merck Animal Health created and tested the survey and then piloted for appropriate branching and potentially ambiguous or missing response options. Results of the testing were incorporated into the final version of the survey. A link to the survey was distributed *via* an email invitation to all members of the American Association of Swine Veterinarians (AASV), and access was made available from December 2, 2020, to January 30, 2021. AASV has ~1,300 members worldwide, but the survey was limited to swine veterinarians currently practicing veterinary medicine in the United States. This sample included all U.S. swine veterinarians, regardless of whether they had been involved in the COVID-19 depopulation event. An incentive to complete the survey was included by informing potential participants that $25 would be contributed to the AASV Foundation for every completed questionnaire. In addition, two reminder emails were sent. The study was categorized as exempt by Colorado State University's Institutional Review Board, and the survey was administered *via* a Qualtrics survey link with branching logic used to display only questions relevant to each participant. The first question was a screening question to ensure respondents were currently practicing swine veterinary medicine.

Veterinarians who self-identified as not currently practicing swine veterinary medicine were eliminated from further analysis. The body of the survey consisted of demographics (i.e., age, race, ethnicity, sex, marital status, number of children), general employment questions (e.g., current position, type of practice), and several questions related to mental health. These included views about mental health, personal experiences, and reported involvement with healthy behaviors (e.g., volunteering, sleeping 8 h/night, socializing with friends).

Additional mental health assessments included The Cantril Self-Anchoring Scale (36), which is comprised of one question that asks participants to place themselves on an 11-step ladder with the worst possible life represented by 0 and best possible life represented by 10. Gallup has used the Cantril Ladder to assess well being in over 150 countries. Scores are categorized into suffering (0–4), Struggling (5, 6), and Thriving (7–10, 37). Participants were also asked to complete The Physician well being Index (38) burnout scale, which is comprised of seven yes/no questions. Participants receive a score from 0–7 (1 point for each item answered “yes”), with scores ≥ 4 indicating a lower quality of life (QOL) and scores <2 reflecting higher QOL.

The Kessler Psychological Distress Scale (39) was used to assess psychological distress. The scale consists of 6 items in which respondents assign a score. The score ranges from 1 (none of the time) to 5 (all of the time) to determine the presence or absence of psychological distress. Significant psychological distress is determined by scores ≥13 (40).

The Brief Resilience Scale (BRS) (41) is a short scale used to measure the ability to bounce back from stress (42). The BRS is positively correlated with optimism, purpose in life, social support, and coping mechanisms. Conversely, it is negatively correlated with pessimism and negative interactions, behavioral disengagement, perceived stress, anxiety, depression, fatigue, and pain (41). The scale consists of six items to which participants are asked to indicate agreement level on a 5 point Likert scale (1 = strongly disagree, and 5 = strongly agree). Cronbach's alpha has been found to range from 0.80 to 0.92 (41).

Next, participants were asked their agreement level on a 5-point scale (1 = strongly disagree 5 = strongly agree) to 14 questions about job satisfaction. Although some of these questions have been asked in previous studies (9, 10), the created factors were unique to this study. Factor analysis was used to create three factors from these questions including intrinsic job factors (α = 0.83), environment (α = 0.66), and benefits/job factors (α = 0.74); explaining 38.8, 10.98, and 8.29% of the variance, respectively (total variance explained was 58.03%).

The next series of questions pertained to the COVID-19 depopulation effort. Participants were asked to indicate their involvement level with depopulation before COVID-19 and their involvement with the COVID-19 depopulation effort. Those who indicated they were involved in the COVID-19 depopulation effort were then asked a series of questions about their involvement, including the period of time involved, an approximate number of pigs depopulated, method(s) of depopulation utilized. Potential distress related to the COVID-19 depopulation was assessed with 13 items to which participants were asked to rate on a 5-point Likert scale from 1 = no distress at all to 5 = extreme stress. Factor analysis was used to construct two factors of COVID-19 depopulation distress that explained 48.5 and 12.36% variance, respectively. These factors were Ethics of care (α = 0.90) and Perceptions of others (α = 0.87).

Those veterinarians involved in the COVID-19 depopulation event were also given select questions from the Event Characteristics Questionnaire (ECQ). The ECQ (43) is designed to assess the impact of major events and consists of 38 items that assess nine categories. This study included four questions for five categories: challenge (amount of stress and anxiety associated with the event), emotional significance (emotional impact of the event); impact (the extent to which one's life has changed due to the event), change in world views (how much one's views have changed due to the event), and social status change (changes in one's social status).

Descriptive statistics were calculated for participant demographics, mental health assessments, and depopulation-related variables. Principal axis factor was used with principal component analysis for extraction and Oblimin with Kaiser Normalization rotation method. The number of factors for Job Satisfaction and COVID-19 depopulation distress were chosen because of the “leveling off” of eigen values on the scree plot. Multiple regression was used to explore predictors of well being, burnout, and psychological distress. Pearson correlation was used to assess the relationship between resiliency [as measured by the Brief Resilience Scale (BRS)] and well being, burnout, and psychological distress. One-way ANOVA was used to explore the relationship between involvement in the COVID-19 depopulation event and well being, burnout, and psychological distress. Lastly, one way ANOVA was used to explore the relationship between method of depopulation used (VSD+ or other methods) on well being, psychological distress, burnout, depopulation distress (ethics of care, perception of others), and ECQ factors (challenge, emotional significance, impact, change in world views, social status change). The significance level (α) was set at a conservative *p* = 0.01 due to the number of analyses, and all tests were two-tailed. Data were analyzed using SPSS (IBM, Armonk, NY, USA).

## Results

### Goal 1: Assess the Mental Health of Swine Veterinarians Compared to the U.S. Veterinary Population, Including Well Being, Quality of Life (QOL), Psychological Distress, Burnout, and Resilience

**Figure 1:**
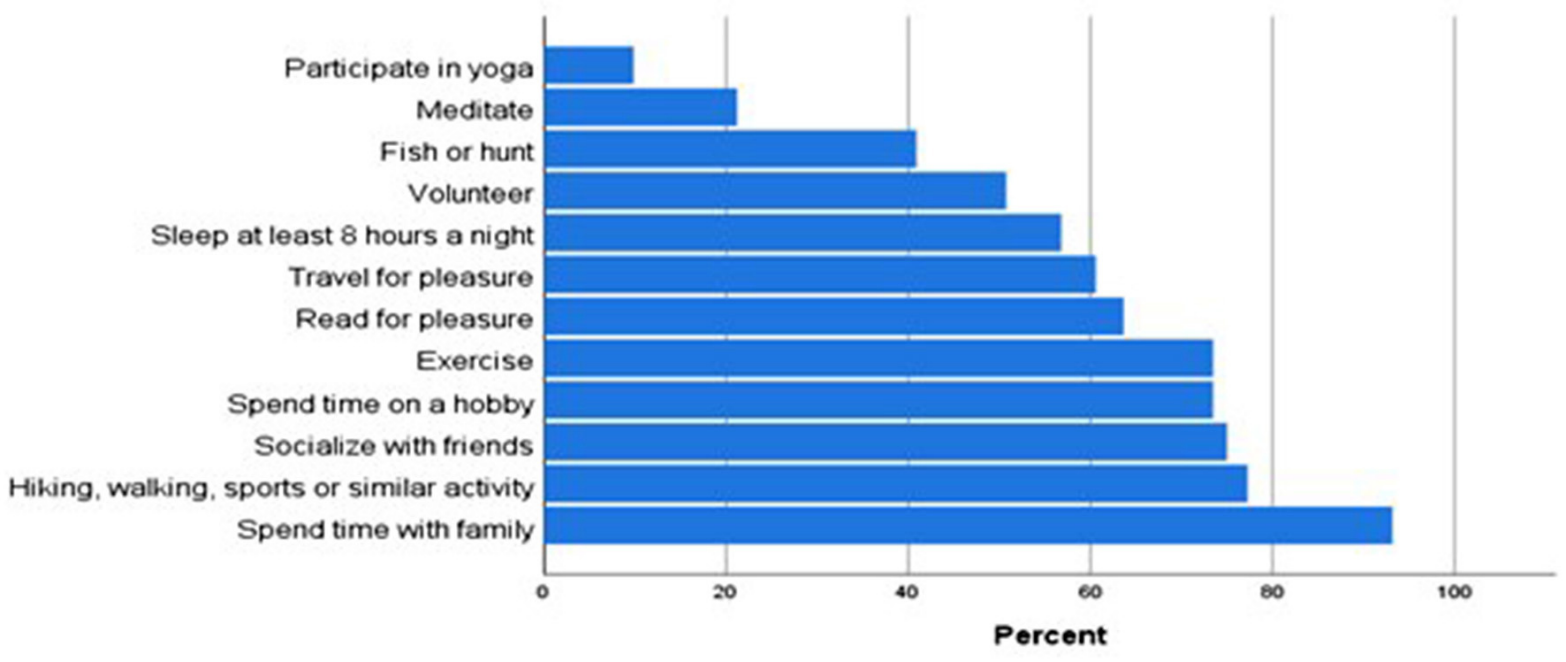
Engagement in healthy behaviors at least sometimes (*n*-132).

A total of 134 participants completed the survey. Because some participants chose not to answer every question, the number of responses for each question was recorded. The sample consisted of 44 (34.1%) female and 85 (65.9%) male (*n* = 129); primarily White (96.2%, 126/131), non-Hispanic or Latino (98.5%, 128/130), married (87.8%, 115/131), with no children (53.4%, 70/131). Ages ranged from 25 to 74. For analyses purposes, age was categorized into two groups: younger than 45 years of age (47.7%, 63/132) and 45 years of age or older (52.3%, 69/132) (Table 1). When asked about frequency in which they participate in several types of healthy behaviors (*n* = 132), the behaviors most frequently endorsed “at least sometimes” include spending time with family (93.2%), hiking, walking or similar activity (77.3%), and socializing with friends (75.0%) (Figure 1).

**Table 1:** Demographics of swine veterinarians that participated in mental health survey.

**Age (132)**	**Younger than 45**	**45 and older**			
	63 (48%)	69 (52%)			
Race (131)	African-American/black	American Indian/Native Alaskan	Asian	LatinX/Hispanic	White
	1 (1%)	1 (1%)	1 (1%)	2 (2%)	126 (96%)
Ethnicity (130)	Hispanic or Latino	Not Hispanic or Latino			
	2 (2%)	128 (99%)			
Sex (129)	Female	Male			
	44 (34%)	85 (66%)			
Marital status (131)	Single	Married/Partner	Divorced		
	15 (12%)	115 (88%)	1 (1%)		
Dependent children (131)	0	1	2	3	4
	70 (53%)	16 (12%)	28 (21%)	15 (12%)	2 (2%)

Job characteristics are summarized in Table 2. The largest number of participants reported their position as owner/co-owner of a veterinary practice (35.8%, 48/132), followed by a staff veterinarian for a livestock production company (24.6%, 33/132). For analysis purposes, positions were categorized into associate/staff (41%), owner (37.1%), and other (22.0%). When asked about practice type, the most common response was swine practice exclusively (39.6%, 53/132). When queried about location, the largest percentage of respondents reported living in Iowa (26.9%), Illinois (13.4%), or Minnesota (11.2%). They reported working an average of 49 h a week, and most were satisfied with the number of hours they work and the number of hours they spend on-call (Table 2). The majority work for companies having five or fewer full-time employees (63.5%, 61/96) and reported making $150,000 or more annually. Most participants reported they would recommend a career in veterinary medicine to a friend or family member (76.6%, 98/128) (Table 2).

**Table 2:** Job characteristics of swine veterinarians participating in mental health survey.

**Current position (132)**	**Associate vet**	**Consultant**	**Owner/co-owner of veterinary practice**	**Owner/co-owner of a swine operation**	**Staff veterinarian for a livestock production company**	**Manager**	**Other**
	21 (16%)	13 (9.7)	48 (35.8)	1 (0.7)	33 (24.6)	5 (3.7)	13 (9.7)
Position	Associate/staff	Owner	Other				
	54 (41%)	49 (37.1)	29 (22.0)				
Practice type	Swine practice (predominant)	Swine practice (exclusive)	Mixed practice (at least 25% swine)	Livestock production or processing company	Other		
	30 (22%)	53 (40%)	14 (10%)	16 (12%)	21 (16%)		
States	IA	IL	MN	NC	SD	IN	KS
	36 (27%)	18 (13%)	15 (11%)	10 (8%)	10 (8%)	9 (7%)	5 (4%)
Hours work per week	Mean (SD)	Median					
	49.44 (13)	51					
Satisfaction with workload (130)	More hours than like	Less hours than like	Satisfied with hours				
	51 (39%)	2 (2%)	77 (59%)				
On-call hours (130)	More on call than like	Less on call then like	Satisfied with on call hours	Don't have on call hours			
	21 (16%)	-	57 (44%)	52 (40%)			
Number of FTEs (96)	0.5–5	5.5–10	10.5–15	≥15.5			
	61 (63%)	13 (14%)	16 (17%)	6 (6%)			
Income (111)	≤ $99,999	$100,000-$149,999	≥$150,000				
	29 (26%)	36 (32%)	46 (41%)				
Recommend veterinary medicine (128)	Yes	No					
	98 (77%)	30 (23%)					

In response to a series of questions about mental health support, the majority indicated they had never received mental health services in the form of outpatient care, overnight care, or prescription medications. Yet, 16.4% indicated they felt they needed mental health counseling but did not get it. When asked why they did not seek treatment, the most common responses were concerns about counseling, taking time off work, and expense/insurance. Next, participants were asked if they had thought about suicide, to which 14 (10.4%) reported yes, 3 (2.2%) in the past 12 months, and 11 (8.2%) prior to the past 12 months. Next, when asked to indicate if they had an employee assistance program that includes mental health, 42.7% said yes, 27.5% said no, and 29.8% did not know. Additionally, when asked if their health insurance covers mental health, the majority either said yes (50.7%) or that they did not know (44.0%) (Table 3).

**Table 3:** Mental health support reported by swine veterinarians (*n* = 134).

	**Yes, in past 12 months**	**Yes, not in past 12 months**	**No**	**Related to depopulation**
Prescription medication for mental health	7 (5%)	9 (7%)	118 (88%)	
Outpatient care for mental health	7 (5%)	20 (15%)	107 (80%)	1
Overnight care for mental health			134 (100%)	
Needed mental health counseling but didn't get it^*^	22 (16%)	9 (7%)	103 (77%)	7
Thought about suicide	3 (2%)	11 (8%)	119 (89%)	1
-Made suicide plans	1/13	5/13	7/13	
-Tried to kill self			6/6	
Contacted suicide hotline	1/14	1/14	12/14	
^*^Why didn't seek treatment:	Concerns about counseling	Issues with taking time off work	Expense/insurance	
	32	17	12	
	Yes	No	Don't know	Don't have health insurance
Employee assistance program that includes mental health	56 (42%)	36 (28%)	39 (30%)	1 (1%)
Health insurance covers mental health	68 (51%)	5 (4%)	59 (44%)	

* *Multiple responses allowed*.

In addition to personal experiences, respondents were asked to indicate their agreement level to several mental health-related questions. The statements with the highest agreement level include “I feel my employer/partners/spouse would support me if I needed to take time off work to seek mental health treatment” (83.5%), and “I have effective ways/methods to handle the stress in my life” (77.4%) (Table 4).

**Table 4:** Attitudes about mental health as reported by swine veterinarians.

	**Agree**	**Neutral**	**Disagree**
I feel comfortable discussing mental health topics with other veterinarians	50%	23%	26%
Veterinarians are supportive of other veterinarians with emotional problems or mental health issues	53%	33%	14%
If needed, I would feel comfortable asking to take time off work to seek mental health treatment	59%	14%	27%
Funding accessible mental health treatment options should be a top priority within the veterinary field	62%	32%	6%
Mental health treatment is accessible to veterinarians who need/want it	63%	27%	10%
I have effective ways/methods to handle the stress in my life	77%	19%	4%
I feel my employer/partners/spouse would support me if I needed to take time off work to seek mental health treatment	84%	7%	10%

#### Mental Health Assessments

##### Candril Ladder Scale Index

The range of scores for The Candril Ladder Index was 1–6, with a mean of 3.25 (SD = 1.19). The majority of participants scored in the thriving category (117, 87.3%), with 17 (12.7%) scoring in the struggling category and none in the suffering (lowest) category.

##### The Physician Well Being Index Burnout Scale

In the current study, Cronbach's alpha for the Physician Well being Index burnout scale was 0.79. The range of values for the seven yes/no questions (1 point for each item answered “yes”) of respondents was 0–7; the mean score for all participants was 2.32 (SD 2.05). The number of scores ≥ 4 (suggesting higher levels of burnout) was 39 (29.2%), and the number of scores <2 (suggesting lower levels of burnout) was 55 (41.0%). The remaining scores (*n* = 40) were >2 but <4.

##### The Kessler 6 Psychological Distress Scale

In the current study, the Cronbach's alpha for the Kessler 6 Psychological Distress Scale was 0.81. The range of values was 1–6, with a mean of 4.67 (SD = 3.17). A total of 4 participants (3.0%) scores ≥13, indicating significant psychological distress.

##### Brief Resilience Scale (BRS)

Cronbach's alpha for the Brief resilience scale (BRS) was 0.87. The mean score was 3.73 (SD = 0.64) with a range of 1–5, with higher scores indicating a higher ability to recover from stress. The BRS (*n* = 133) was negatively correlated with Candril's Ladder Index (-0.448, *p* < 0.001), the Physician Well being Index Burnout Scale (-0.404, *p* < 0.001), and the Kessler 6 Psychological Distress Scale (-0.426, *p* < 0.001).

##### Event Characteristics Questionnaire (ECQ)

The Event Characteristics Questionnaire (ECQ) was only given to participants involved in the COVID-19 related depopulation effort. Four questions were asked about five categories; “Challenge,” which refers to the event itself, “Impact,” “Emotional Significance,” “Change in World Views,” and “Change in Social Status”; all referring to the perceived consequences. Reliability for each of these categories in the current study was: challenge (α = 0.91), emotional significance (α = 0.91); impact (α = 0.83), change in world views (α = 0.88), and social status change (α = 0.86). The mean score for each category was: Challenge = 2.50 (1.22); Emotional significance = 2.59 (1.19), Impact = 1.78 (0.85), Change in World Views = 2.36 (1.06) and Change in Social Status = 1.25 (0.62).

#### Job Satisfaction Scale

The suitability of the 14 questions about job satisfaction was assessed for factor analysis. All of the questions were deemed suitable, and Principal Components Analysis was used to identify and compute composite scores for the factors underlying job satisfaction. Principal axis factor was used with principal component analysis for extraction and Oblimin with Kaiser Normalization rotation method. The three factor solution, which explained 58.0% of the variance, was preferred because of the “leveling off” of eigen values on the scree plot after three factors. Initial eigenvalues indicated that the first three factors explained 38.8, 10.98, and 8.29% of the variance, respectively. Factor 1, Intrinsic Job Factors, consists of six items, including “I am often intensely focused on my work and time goes by quickly” and “I am enjoying the work that I do.” The second factor, Environment, consists of four items, including, “My supervisor treats me with respect and values my work.” The third factor, Job Factors and Benefits, is comprised of four factors, including “I have flexible work hours and can determine the amount of work I do.” (Tables 5–7).

**Table 5:** Job satisfaction principal component analysis.

**Total variance explained**			
**Component**	**Initial eigenvalues**		
	Total	% of Variance	Cumulative %
^1^	5.43	38.76	38.76
^2^	1.54	10.98	49.75
^3^	1.16	8.29	58.03
Extraction Method: Principal Component Analysis.			

1 *Intrinsic job factors*.

2 *Environment*.

3 *Job factors and benefits*.

**Table 6:** Job satisfaction principal component analysis matrix.

	**Component**		
	**^1^**	**^2^**	**^3^**
I am often intensely focused on my work, and time goes by quickly	0.854		
I am enjoying the work that I do	0.802		
My work makes a positive contribution to other people's lives	0.668		
I feel invigorated after working with clients	0.666		
I often learn something new at work	0.621		
I am invested in my work and take pride in doing a good job	0.577		
I am satisfied with my position and promotion opportunities	0.499		
I have a good balance between my work life and my personal life		−0.742	
I have flexible work hours and can determine the amount of work I do		−0.691	
I think that I am paid fairly and adequately for my work		−0.499	
A co-worker or supervisor is creating a negative work environment			0.691
My supervisor treats me with respect and values my work			−0.691
I have a warm, friendly, and supportive relationship with my co-workers			−0.684
I decide how I structure my work and how the work gets done			−0.641
Extraction Method: Principal Component Analysis. Rotation Method: Oblimin with Kaiser Normalization.			

1 *Intrinsic job factors*.

2 *Environment*.

3 *Job Factors and benefits*.

**Table 7:** Job satisfaction factors.

**Factor 1 Intrinsic Job Factors (α = 0.83)**
**•I am enjoying the work that I do**
**•My work makes a positive contribution to other people's lives**
**•I feel invigorated after working with clients**
**•I am invested in my work and take pride in doing a good job**
**•I often learn something new at work**
**Factor 2—Environment (α = 0.66)**
**•A co-worker or supervisor is creating a negative work environment**
**•My supervisor treats me with respect and values my work**
**•I have a warm, friendly, and supportive relationship with my co-workers** **•I decide how I structure my work and how the work gets done**
**Factor 3 Job Factors and Benefits (α = 0.74)**
**•I have flexible work hours and can determine the amount of work I do**
**•I have a good balance between my work life and my personal life**
**•I think that I am paid fairly and adequately for my work**
**•I am satisfied with my position and promotion opportunities**

#### Impact of Work Factors, Gender, and age on Mental Health

To assess the impact of work factors [job satisfaction (intrinsic factors, benefits, environment), salary, hours worked, position (associate/owner/other)], in addition to demographics (gender, and age) on well being (as measured with the Candril Ladder Index), we conducted linear regression. The overall model was significant (*F*_(8)_ = 13.36, *p* < 0.001), with an *r*^2^ of 0.517. The only significant factor of well being in the model was job satisfaction—intrinsic factors (B = 1.03 *p* < 0.001) (Table 8).

**Table 8:** Results of the multiple linear regression model predicting well being as a function of job factors, gender, and age.

**ANOVA**						
**Model**	**Sum of squares**	**df**	**Mean squares**	**F**	**Sig**.	
Regression Residual Total	81.26 76.03 157.28	8 100 108	10.16 0.76	13.36	<0.001
Coefficients ^*^ (Dependent Variable: Well being)						
Variable			Coefficient (B)	Std. Error	t	Sig.
(Constant)			1.840	0.687	2.68	0.009
**Job satisfaction factors—Intrinsic**			**1.029**	**0.175**	**5.89**	**<** **0.001**
Job satisfaction factors—Benefits			0.130	0.130	1.00	0.318
Job satisfaction factors—Environment			0.156	0.134	1.17	0.246
Salary			−0.104	0.113	−0.92	0.359
Position			0.129	0.119	1.08	0.283
Number of hours			0.049	0.221	0.22	0.825
Gender			−0.490	0.201	−2.44	0.016
Age			−0.343	0.193	−1.77	0.079

* *Significant predictors are shown in Bold*.

Similarly, we conducted linear regression to assess the impact of work factors, [job satisfaction (intrinsic factors, benefits, environment), salary, hours worked, position (associate/owner/other)], and demographics (gender, and age) on burnout. While the overall regression model was significant (*F*_(8)_ = 4.86, *p* < 0.001), *r*^2^ = 0.280), none of the factors significantly predicted burnout. The same results were seen when assessing the impact of work factors, age, and gender on psychological distress. The linear regression model was significant (*F*_(8)_ = 3.97, *p* < 0.001), *r*^2^ = 0.241, but none of the factors significantly predicted psychological distress.

#### Goal 2: Identify Coping Methods Utilized by Swine Veterinarians for Improved Well Being

##### Impact of Healthy Behaviors on Mental Health

The impact of healthy behaviors on well being was analyzed with linear regression. While the overall model was significant (*F*_(12)_ = 2.71, *p* = 0.003), with an *r*^2^ of 0.135, no behaviors had a significant effect. The linear regression model used to determine the predictive value of healthy behaviors on burnout was also significant (*F*_(12)_ = 2.45, *p* = 0.007, *r*^2^ = 0.198) and the one significant predictor was sleeping at least 8 h a night (B = −0.92 *p* < 0.009). Lastly, the regression equation assessing the impact of healthy behaviors on psychological distress was significant (*F*_(12)_ = 3.17, *p* = 0.001, *r*^2^ = 0.242). Significant predictors of decreased psychological distress included socializing with friends (B = −1.72 *p* = 0.013), spending time with family (B = −3.15 *p* = 0.003) and sleeping at least 8 h a night (B = −1.72 *p* < 0.001) (Table 9).

**Table 9:** Results of the multiple linear regression model predicting psychological distress as a function of healthy behaviors.

**ANOVA**					
**Model**	**Sum of squares**	**df**	**Mean squares**	***F***	**Sig**.
Regression Residual Total	320.79 1002.20 1322.99	12 119 131	26.73 8.42	3.17	<0.001
Coefficients ^*^ (Dependent Variable: psychological distress)					
Variable	Coefficient (B)	Std. Error	t		Sig.
(Constant)	10.292	1.256	8.192		0.000
Exercise	−0.074	0.699	−0.105		0.916
Participate in yoga	−0.206	0.941		−0.219	0.827
**Socialize with friends**	–**1.719**	**0.678**	–**2.534**		**0.013**
Meditate	0.547	0.674	0.812		0.418
Read for pleasure	−0.534	0.617	−0.866		0.388
Travel for pleasure	0.049	0.619	0.079		0.937
Fish or hunt	−0.877	0.529	−1.659		0.100
Volunteer	−0.375	0.609	−0.616		0.539
Spend time on a hobby	−0.551	0.621	−0.887		0.377
**Spend time with family**	–**3.145**	**1.047**	–**3.002**		**0.003**
Hiking, walking, sports or similar activity	1.038	0.742	1.399		0.164
**Sleep at least 8 h a night**	–**1.719**	**0.525**	–**3.274**		**0.001**

* *Significant predictors are shown in Bold*.

#### Goals 3 and 4: Compare the Mental Health of Swine Veterinarians Involved With Mass Depopulation Events With Swine Veterinarians Who Were Not Involved in Mass Depopulation and Measure the Potential Impact of Depopulation Methods on Swine Veterinarians' Mental Health

##### COVID-19 Depopulation

Participants were asked to indicate their involvement with depopulation efforts prior to COVID-19 and their involvement with the pandemic-related depopulation. Prior to COVID-19, nearly 40% indicated they had been aware of depopulation events taking place in the industry but had not been involved in any way, and 35% had been personally involved. For the COVID-19 depopulation effort, 41% indicated they were not involved. For analysis purposes, participants were divided into two groups; any type of involvement with the COVID-19 depopulation effort or no involvement (Table 10).

**Table 10:** Experience with depopulation efforts before COVID-19 and as a result of COVID-19.

	**Prior to COVID-19 depopulation**	**COVID-19 depopulation**	**COVID-19 depopulation involvement**
I was not aware of any depopulation taking place in the industry		3 (2.2%)	No
I have been aware of depopulation events taking place in the industry but have not been involved in any way	53 (39.6%)	52 (38.8%)	No
I have work(ed) for a veterinary practice or swine operation that depopulated pigs but have not been personally involved	13 (9.7%)	13 (9.7%)	Yes
I have consulted on the need to depopulate but have not been directly involved in selecting the method or depopulating	18 (13.4%)	23 (17.2%)	Yes
I have recommended, or decided on, the method for my client or employer to depopulate pigs	24 (17.9%)	30 (22.4%)	Yes
I have been personally involved in depopulating pigs	47 (35.1%)	40 (29.9%)	Yes

Those who indicated involvement with the COVID-19 depopulation effort were asked to indicate the type(s) of methods used (*n* = 78). These methods were divided into two categories: AVMA preferred (carbon dioxide, penetrating captive bolt, non-penetrating captive bolt, electrocution, gunshot, and anesthetic overdose) and AVMA permitted under constrained circumstances [sodium nitrite and ventilation shutdown plus (VSD+)]. The most common method of depopulation used was carbon dioxide (48.7%), followed by penetrating captive bolt (32.1%), and VSD+ (32.05%) (Table 11). Participants were also asked how long they were involved in the depopulation effort and the total number of pigs depopulated. The most common amount of time was more than 4 weeks (57.5%). For further analysis, two categories for time were created: ≤ 4 weeks (31, 42.5%) and >4 weeks (42, 57.5%). The responses to the question about the total number of pigs involved were also used to create two categories: <5,000 pigs (36, 52.7%) and ≥5,000 pigs (31, 46.3%).

**Table 11:** Method of depopulation utilized by veterinarians. More than one method could be selected by the veterinarian.

	**AVMA preferred**	**AVMA permitted under constrained circumstances**
Carbon dioxide	38 (48.7%)	
Penetrating captive bolt	25 (32.1%)	
Sodium nitrite		21 (26.9%)
Ventilation shutdown Plus (VSD+)		25 (32.05%)
Non-penetrating captive bolt	14 (17.9%)	
Electrocution	14 (17.8%)	
Gunshot	13 (16.7%)	
Anesthetic overdose	1 (1.3%)	

##### Stress Related to the Depopulation Effort

A total of 13 questions were asked to assess stress related to the depopulation effort, 12 of which were deemed suitable for factor analysis. Principal axis factor was used with principal component analysis for extraction and Oblimin with Kaiser Normalization rotation. Two factors, which explained 60.86% of the variance, was chosen because of the “leveling off” of eigen values on the scree plot after two factors. Initial eigenvalues indicated that the first two factors explained 48.5 and 12.36% variance, respectively. Factor 1, Ethics of Care, included eight items and had a Cronbach's alpha of 0.90. Factor 2, Perceptions of Others, included four items and had a Cronbach's alpha of 0.87 (Table 12).

**Table 12:** Pattern matrix for stress related to depopulation participation by the veterinarian.

	**Component**	
	**Factor 1—ethics of care**	**Factor 2—perceptions of others**
Participating in killing the animals	0.923	
The idea of depopulating healthy animals	0.910	
Making the decision whether or not to depopulate	0.844	
Selecting the method to use for depopulation	0.690	
Seeing the emotional toll on employees involved in depopulation	0.630	
Seeing the emotional toll on clients or company management caused by depopulating	0.589	
Disposal of depopulated animals	0.588	
Economic hardship on my client or my employer	0.439	
Public criticism of depopulation methods		0.940
Public criticism of depopulation		0.848
Criticism from veterinary colleagues for depopulation method used		0.847
Criticism from family, friends, or neighbors for killing healthy animals		0.606

*Extraction Method: Principal Component Analysis*.

*Rotation Method: Oblimin with Kaiser Normalization*.

##### Depopulation Involvement and Mental Health

The relationships between involvement in the COVID-19 depopulation event (yes/no) and well being, psychological distress, burnout, depopulation distress (Ethics of Care, Perception of Others), ECQ factors (Challenge, Emotional Significance, Impact, Change In World Views, Social Status Change) were assessed with a one-way ANOVA. The only significant relationship was between depopulation involvement and burnout (*F*_(132)_ = 12.41, *p* = 0.001), with higher levels of burnout reported by those involved in the depopulation effort.

##### Depopulation Method

The relationship between method of depopulation used (VSD+ or other methods) and well being, psychological distress, burnout, depopulation distress (ethics of care, perception of others), ECQ factors (challenge, emotional significance, impact, change in world views, social status change) was assessed with a one-way ANOVA. The method of depopulation significantly impacted depopulation distress [Ethics of Care (*F*_(76)_ = 7.63, *p* = 0.007), Perception of Others (*F*_(76)_ = 20.77, *p* < 0.001) and Burnout (*F*_(132)_ = 17.02, *p* < 0.001) (Table 13).

**Table 13:** ANOVA table assessing the relationship between depopulation method (VSD+ or other methods) and well being, psychological distress, burnout, depopulation distress (ethics of care, perception of others), and ECQ factors.

**ANOVA**					
	**Sum of squares**	**df**	**Mean square**	***F***	**Sig**.
Well being	0.135	132	0.135	0.094	0.759
**Depopulation distress—ethics of care**	**4.819**	**76**	**4.819**	**7.631**	**0.007** ^*^
**Depopulation distress—perception of others**	**19.676**	**76**	**19.676**	**20.770**	**<0.001**
ECQ—challenge	4.590	**76**	4.590	3.190	0.078
ECQ—emotional significance	4.431	**76**	4.431	3.209	0.077
ECQ—Impact	1.591	**76**	1.591	2.241	0.139
ECQ- change in world views	6.522	**76**	6.522	6.211	0.015
ECQ- social status change	0.147	**76**	0.147	0.380	0.540
Psychological distress	39.370	131	39.370	4.016	0.047
**Burnout**	**63.651**	**132**	**63.651**	**17.023**	**<0.001**

* *Significant predictors are shown in Bold*.

## Discussion

This study was designed to better understand the mental health needs related to well being, quality of life (QOL), psychological distress, burnout, and resilience of all U.S. swine veterinarians, both those who applied mass depopulation methods during COVID-19 and those who did not. Additionally, we assessed the coping methods utilized by swine veterinarians for improved well being. We have used these results to create recommendations for intervention strategies and supportive services to assist veterinarians in future mass depopulation events.

Numerous studies have documented the increased risk of anxiety, depression, and suicidal thoughts and lower levels of positive mental well being among veterinarians in the United States (44–46), the United Kingdom (47–49), and Australia (50, 51). A recent study by Perret (6) found that Canadian veterinarians had higher levels of perceived stress, burnout, depression, anxiety, compassion fatigue, and suicidal ideation than the general population. Burnout, a psychological syndrome comprised of emotional exhaustion, depersonalization, and a sense of reduced personal accomplishment (52), was also noted as a significant area of concern in a recent study of U.S. veterinary house officers (53). Both compassion fatigue and burnout result from external and internal stressors commonly found in veterinary medicine (4).

The Merck Animal Health Veterinary Wellbeing Studies (9, 10) found that veterinarians have lower well being than the general population but did not find an overall difference in serious psychological distress. However, upon deeper analysis, they found that young and female veterinarians were more likely to suffer from serious psychological distress and lower well being levels than older and male veterinarians. Additionally, they found that food animal veterinarians had higher levels of well being than other types of veterinarians and the general public. Similarly, the current study found that the majority of swine veterinarians report high levels of well being. Utilizing The Candril Ladder Index, we found that 87% of participants scored in the thriving category, 13% in the struggling category, and no one in the lowest (suffering) category.

Additionally, only 3% of participants reported significant psychological distress as measured by The Kessler 6 Psychological Distress Scale. Similar scores for well being and psychological distress among large animal veterinarians were reported by Volk (9, 10). When assessing burnout in the current study, the mean score of all participants was 2.32, comparable to a score of 2.4 for swine veterinarians in the Merk study (10). This translates into 29% of participants reporting at least moderate levels of burnout. It is important to note that there was a significant relationship between participants' involvement in the COVID-19 depopulation effort and the method of depopulation utilized on reported levels of burnout.

Numerous factors are related to high stress and negative mental health among veterinarians. It has been suggested that some personality traits might increase the risk of anxiety, depression, and burnout among veterinarians. These traits include perfectionism, neuroticism, and a preference to work with animals rather than people (54). External factors that can impact mental health include both work and personal issues. Work factors include hours worked, client expectations, relationships with colleagues, complaints, and litigation risks (55). Personal factors include personal finances, career concerns, and difficult life circumstances (49, 56, 57). In the current study, we found that higher endorsement of intrinsic job factors (e.g., “I am enjoying the work that I do,” “I often learn something new at work”) predicted higher levels of well being.

Because healthy lifestyle behaviors can positively impact mental health, in addition to questions about psychological support services, participants were asked to report their engagement in healthy behaviors. Most participants reported engaging in various self-care habits, with the most common ones including spending time with family, hiking, walking, or similar activity, socializing with friends, and spending time on a hobby. The percent of participants who reported engaging in these activities was higher than that reported by Volk (9). When assessing the impact of these healthy lifestyle behaviors, we found that sleeping at least 8 h a night positively affected burnout and psychological distress. Other behaviors that were found to mitigate psychological distress include socializing with friends and spending time with family.

One serious consequence of negative mental health that has garnered increased attention is suicide. It has been noted that veterinarians and veterinary technicians are at increased risk for suicide, suicide attempts, and suicidal ideation (57, 58). Furthermore, a 2014 survey of U.S. veterinarians found that female veterinarians consider suicide more often than males; but both male and female veterinarians consider suicide more often than the general population (45). Female veterinarians in clinical roles are 3.4 times more likely to die by suicide than the general population (59). Other studies have echoed these results, finding that female veterinarians have a higher prevalence of risk factors for suicide and higher suicide rates than male veterinarians or the general population (46, 47, 54, 60, 61). In Volk (10), 10% of swine veterinarians indicated they had thought about suicide; a similar percentage (10%) of respondents reported suicide ideation in the current study.

One unique factor of veterinary medicine is euthanasia. Euthanasia has been defined as a moral stressor, qualitatively different from other types of workplace stressors (24). Several studies have documented the negative effects of euthanasia on veterinary professionals (62–64). Involvement with euthanasia has been shown to result in compassion fatigue, stress-related somatic complaints, work-family conflict, and lower levels of job satisfaction (31, 63–65).

The term “caring–killing paradox,” first coined by Arluke (65), describes the moral challenge of understanding the necessity of euthanasia, but at the same time, having compassion and feelings toward animals. Holding both moral views simultaneously can be challenging at best. Accepting the need to euthanize does not remove the negative feelings many feel toward killing animals. This internal conflict can cause identity-threatening challenges and adversely affects job satisfaction, employee turnover rates, and physical and mental health (63, 66, 67). Further, the negative impact of euthanasia appears greater when those involved are attached to the animals (31, 66). Public perception and the stigma associated with euthanasia can exacerbate these challenges (67). One study exploring the negative emotional impacts of euthanasia within animal shelter employees found a positive effect in following best practices (63).

Depopulation is a unique form of euthanasia typically conducted due to disease (68). Several studies have shown that veterinarians, farmers, producers, and others involved in killing many animals suffer distress and have increased risks of mental health problems (16, 69, 70). Whiting and Marion (16) noted that the reasoning and rationale behind the decision to depopulate are often not sufficient to prevent mental health challenges.

For some people, the stress associated with performing euthanasia or depopulation can cause perpetration-induced traumatic stress (PITS), a type of Post Traumatic Stress Disorder (PTSD) (26, 29). PITS was first identified in American veterans of the Vietnam war, where it was discovered that those who killed humans, or believed their actions resulted in human death, were significantly more likely to experience PTSD than those who had not directly participated in human killing but had witnessed it (25). The mental stress and negative mental health outcome related to the actual perpetration of lethal violence has been confirmed in war and a wide array of settings, including euthanasia and slaughter (26, 71–74). PITS can create anxiety, panic, depression, and a sense of disintegration and dissociation (25). The diagnostic symptoms of PTSD include a significant impact in daily life from one or more intrusion symptoms (e.g., nightmares) linked to the traumatic event, constant avoidance of anything related to the traumatic event, significant changes in mood or cognition associated with the traumatic event, and significant reactivity and alertness.

The current study found a significant relationship between depopulation involvement and burnout, with higher levels of burnout reported by those involved in the depopulation effort. Previous studies have noted the negative psychological impact of participating in depopulation efforts, including a recent analysis by Vroegindewey (75), who reported that 50% of respondents reported immediate behavioral health issues and 32% reported still having symptoms six months after deployment. Furthermore, the depopulation method had a significant impact on burnout and depopulation distress in the form of Ethics of Care and Perception of Others. The distress associated with Ethics of Care concerns the impact on all those involved: the animals as well as the clients, producers, and all those participating in the depopulation event. The decision to depopulate was based on necessity, but as noted earlier, understanding the rationale does not always mitigate the negative emotional effect. The caring-killing paradox is clearly present in these difficult decisions. The distress from the perception of others involves the criticism from the public, friends, family, and colleagues. The anguish of being forced to make difficult depopulation choices was often compounded by a lack of understanding by those not involved. Creating better public messaging to help mitigate this negative compounding effect will be critical in future depopulation efforts.

### Mental Health Services

When asked their opinions about mental health services, between 50 and 65% of participants agreed with statements indicating a willingness to seek support and the importance of mental health services being available to those who need it. However, this leaves nearly half of respondents not feeling comfortable discussing mental health issues or feeling that veterinarians do not support those who need mental health services. Furthermore, only a minority of veterinarians reported receiving mental health services. For example, 80% of respondents indicated they had not received outpatient mental health support, of which 16% reported feeling they needed it but did not obtain it. The reasons for not acquiring this care fell primarily into three categories: concerns about counseling itself (i.e., did not believe it would be effective), concerns about taking time off work, and financial concerns. To that end, only 42% of respondents reported their employee assistance program includes mental health; 30% did not know if their program included such benefits. Similarly, only 51% of respondents said their health insurance covers mental health, and 44% did not know.

Other studies have found that veterinarians have more negative attitudes about mental health treatment and mental illness than the general US population. It is disturbing to note that Nett (45) found that veterinarians experiencing serious psychological distress were even less likely to agree that people are caring toward persons with mental illness than those not experiencing distress. Similar negative views were reported by Kassem (2), who found that negative attitudes toward mental illness were common in the veterinary profession, with males having more negative attitudes toward the effectiveness of treatment for mental illness than females. It has been noted that since veterinary leadership is currently overrepresented by males, a negative attitude toward the effectiveness of treatment for mental illness might translate into less support for these services (2).

### Limitations

As with any research, our study has certain limitations. Even though this survey was intended for all U.S. swine veterinarians, it is possible that because a portion of the survey was about the COVID-19 depopulation event, some veterinarians who were not involved in the depopulation event did not complete the survey. It is also possible that a subsection of those involved in the depopulation did not want to answer questions about it. Yet, our sample appears to be representative of U.S. swine veterinarians. Additionally, the survey asked for some personal mental health information that some participants may not have wanted to share. The fact, however, that our results mirror earlier research on the prevalence of psychological distress, burnout, and suicide ideation among veterinarians suggests that participants responded honestly. Lastly, it is important to note that this survey marks one moment in time, and caution about generalizing to other time periods is warranted. Future qualitative and quantitative research is needed to further develop our understanding of swine veterinarians' mental health needs, not only for the next depopulation effort but in everyday practice.

## Conclusion

This research used a survey to garner 134 responses from the AASV membership within the United States to identify the emotional impact of the depopulation crisis during the 2020 COVID-19 events. The survey considered AASV veterinarians involved with depopulation events and those not involved in depopulation events. Survey analysis indicated that the scale and scope of swine veterinary respondents' behavioral health issues are significant, and the findings indicate that further study and actions to improve mental health outcomes associated with depopulation are warranted.

### Recommendations

#### Goal 5: Based on the Study Results, Make Recommendations for Intervention Strategies and Supportive Services to Assist Veterinarians in Future Mass Depopulation Events

Actions to address the mental health issues of veterinarians who are faced with involvement in depopulation efforts:

1. Organizational mental health training: AVMA, in conjunction with species-based organizations (AASV, American Association of Bovine Practitioners, American Association of Avian Pathologists, American Association of Small Ruminant Practitioners, etc.), utilize existing mental health training programs (76–78). Additionally, enhance recognition, discussion, and application of mental health programs at all organizational meetings. Finally, the reduction and eventual removal of mental health stigma must occur. Proof that the stigma was evident for swine veterinarians in this study came with 47% of veterinarians indicating there may not be support from other veterinarians when faced with emotional problems or mental health issues.
2. National governmental training response: United States Department of Agriculture, APHIS, and State Veterinarians should further expand the mental health training available through the Foreign Animal Disease Preparedness and Response Plan (FAD PReP) (79) NAHEMS Guidelines: Health and Safety. In addition, since the depopulation events caused by the COVID-19 infrastructure disruption fell outside the federal and state authority to respond, the guidelines should be revised to encompass livestock disasters of all types. In doing so, mental health training and resources can be available to veterinarians called into a mass depopulation response. The guidelines should encompass training, protocols, and resources to support veterinarians involved in mass depopulation before, during, and after the response.
3. Veterinary college curriculum: Veterinary curricula need to be expanded to address behavioral health for veterinarians, veterinary technicians, support staff, and animal owners involved in mass depopulation events, perhaps as part of the “Live-long learning concepts” course material (80). Further training should encompass the killing of the animal and veterinary ethical dilemmas, owner/producer interaction skills, and resources for self-care. Additional training for veterinary students on resiliency could be incorporated into existing courses during discussions of ethical dilemmas, euthanasia, humane endings, and mass depopulation (81).
4. Mental Health Experts: Training for mental health experts on issues specific to mass depopulation, human-animal bond, the caring-killing paradox, and PITS are essential. The mental health expert must understand the mass depopulation situation, methods, and outcomes before trust with the veterinary community is built and assistance can be provided [(82), September 15, 2020]. Mental health treatment was not easily accessible to those who needed or wanted it. Additionally, 62% supported funding to make mental health treatment options a top priority within the veterinary field.
5. Health Insurance: Veterinarians and their health insurance providers must recognize mental health as important as physical health. Provision of employer assistance programs (EAP) and mental health coverage programs must be prioritized. As documented in this study, 57% of veterinarians did not have an EAP program (27.5%) or didn't know if they did (29.8%). Equally alarming, close to 50% of veterinarians did not have health insurance coverage for mental health (5%) or did not know if their insurance covered mental health (44%).
6. Research needed: Additional funding and subsequent research are needed to understand the underlying risk factors for people involved, best practices to build resilience, best programs for support/response, and methods to reduce mental health impact associated with mass depopulation. The research should include all food animal species and the associated personnel (veterinarians, veterinary technicians, animal caretakers/owners, ancillary support personnel, and the affected communities).
7. Transparent communication, collaboration, and peer support: Two critical areas the veterinary profession must consider to mitigate the emotional stress for veterinarians who must perform mass depopulation are factors associated with the ethics of care (concerns for all those involved) and the perception of others. The stress impact on veterinarians from the “ethics of care” and the “perception of others” spotlights the improvement needed by the veterinary profession, the food animal organizations (professional and non-governmental), and academic scientists to communicate the science and necessity of mass depopulation in a timely and coordinated manner to all involved parties and the public.

In summary, this research provides evidence of the emotional impact of conducting mass depopulation of swine on veterinarians during the COVID-19 infrastructure disruption of 2020. Furthermore, conducting mass depopulation and the subsequent judgment from the public and veterinary peers significantly affected the swine veterinarians. To address these challenges will take focus, effort, and action by the veterinary profession, mental health professionals, and food animal organizations.

## Data Availability Statement

The raw data supporting the conclusions of this article will be made available by the authors, without undue reservation.

## Ethics Statement

The studies involving human participants were reviewed and approved by Colorado State University. The patients/participants provided their written informed consent to participate in this study.

## Author Contributions

AB and LK were involved with all elements of this study including study design, data analysis and manuscript preparation. All authors contributed to the article and approved the submitted version.

## Funding

The study was funded by a gift from Merck Animal Health. The funding source did not have involvement beyond the primary authors' active participation.

## Conflict of Interest

AB was employed by the company Merck Animal Health. The remaining author declares that the research was conducted in the absence of any commercial or financial relationships that could be construed as a potential conflict of interest.

## Publisher's Note

All claims expressed in this article are solely those of the authors and do not necessarily represent those of their affiliated organizations, or those of the publisher, the editors and the reviewers. Any product that may be evaluated in this article, or claim that may be made by its manufacturer, is not guaranteed or endorsed by the publisher.
